# Likelihood analysis of small field polynomial models of inflation yielding a high Tensor-to-Scalar ratio

**DOI:** 10.1371/journal.pone.0215287

**Published:** 2019-04-24

**Authors:** Ira Wolfson, Ramy Brustein

**Affiliations:** Department of physics, Ben-Gurion University of the Negev, Beer-Sheva, Israel; Aarhus University, DENMARK

## Abstract

Inflationary potentials, with Planckian field excursions, described by a 6th degree polynomial are studied. We solve the Mukhanov-Sasaki equations exactly and employ a probabilistic approach as well as multinomial fitting to analyse the results. We identify the most likely models which yield a tensor-to-scalar ratio *r* = 0.01 in addition to currently allowed Cosmic Microwave Background (CMB) spectrum and observables. Additionally, we find a significant inter-dependence of CMB observables in these models. This might be an important effect for future analyses, since the different moments of the primordial power spectrum are taken to be independent in the usual Markov chain Monte Carlo methods.

## 1 Introduction

**Fig 1:**
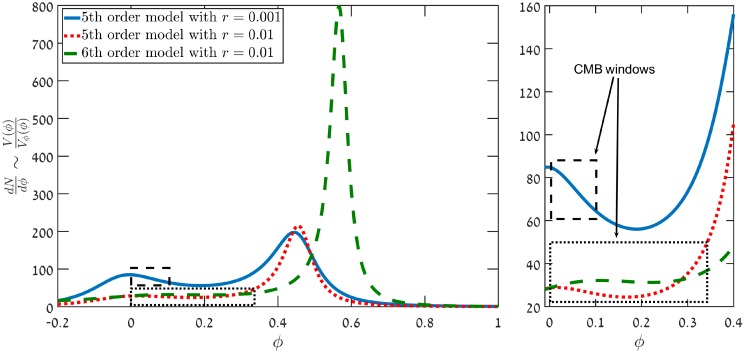
A graph depicting $-1/\sqrt{2\epsilon }∼\frac{V}{V_{\phi }}$ as a function of the inflaton *ϕ* for two fifth-order polynomial models, and a sixth-order polynomial model. For a fifth-order polynomial model with *r* = 0.001 (Blue line) the CMB window width is ∼8 e-folds, while the field changes by about Δ*ϕ* ∼ 0.1. Most of the e-folds are generated when *ϕ* reaches ∼0.4. When *r* is increased the CMB window widens, and approaches the e-fold generating peak (Red dots). While marginally affecting the CMB window width, the introduction of an additional coefficient, *a*_6_, allows to shift the peak to higher values of *ϕ*, thereby decoupling the CMB window and the e-fold generating peak (Green dash).

In a previous article [1], a class of small field inflationary models which are able to reproduce the currently measured Cosmologic Microwave Background (CMB) observables, while also generating an appreciable primordial Gravitational Wave (GW) signal were studied. The existence of such small field models provides a viable alternative to the large field models that generate a high Tensor-to-Scalar ratio. Our exact analysis was shown to give accurate results [1]. Models which yield Tensor to Scalar ratio (*r*), of less than *r* ≲ 0.003 were previously studied in [1]. The initial study demonstrated a significant difference between analytical Stewart-Lyth [2, 3] estimates and the exact results. This result should be confronted with analyses such as in [4] where the Stewart-Lyth expression is relied upon, and [5] in which the authors use a Green’s function approach and perturbation theory, but assume the log of the input is well behaved. Our method extends and improves the method of the model building technique employed in [6, 7]. Previous analytical work [8–10] has shown that a fourth-order polynomial potential is sufficient to generate a high tensor-to-scalar ratio, even up to *r* ≳ 0.1. However, it was hard to realize this numerically. It was discovered in [1], that a fifth-order polynomial potential was required for generating 0.001 ≲ *r* ≲ 0.003. Furthermore a sixth-order polynomial seems to be required for a tensor-to-scalar ratio greater than *r* ≳ 0.003. A simple explanation is offered by observing (see Fig 1) that increasing *r* by factor ∼ 10, causes the e-folds per field excursion generated at the CMB window to decrease by a factor of ∼ 3. This means widening the CMB window, and losing the decoupling between the CMB window and the e-fold generating peak. Adding the 6th coefficient pushes the peak from *ϕ* ∈ [0.4, 0.5], to higher values of *ϕ* and decouples these regions.

### 1.1 Conventions

In this article we follow the conventions of [11]. The Primordial Power Spectrum (PPS) is given by:

$$P_{k}=A_{s}{\left(\frac{k}{k_{0}}\right)}^{n_{s}-1+\frac{\alpha_{s}}{2}\text{log}(\frac{k}{k_{0}})+\frac{\beta_{s}}{6}{\left(\text{log}(\frac{k}{k_{0}})\right)}^{2}}\;.$$ (1)

Our conventions are:

$$n_{s}-1=\frac{\partial \text{log}P_{k}}{\partial \text{log}(k)},$$ (2)

$$\alpha_{s}=\frac{\partial^{2}\text{log}P_{k}}{\partial \text{log}{\left(k\right)}^{2}},$$ (3)

$$\beta_{s}=\frac{\partial^{3}\text{log}P_{k}}{\partial \text{log}{\left(k\right)}^{3}}.$$ (4)

The PPS is expanded about the pivot scale *k*_0_. The pivot scale is usually set a-posteriori, at the scale in which the parameters {*n*_*s*_ − 1, *α*_*s*_, *β*_*s*_} are minimally dependent [12–14], in Planck + BICEP2 data analyses it is usually set at *k*_0_ = 0.05 *hMpc*^−1^.

## 2 Inflationary models

The small field models previously studied in [1] yielded results that are consistent with observable data up to values of *r* ≃ 0.003. While these values agree with the current limits on *r* set by Planck [15, 16], we are interested in studying models with higher *r*. The study of small field models is motivated by their appearance in many fundamental physics frameworks, effective field theory, supergravity [17] and string theory [18] in successive order of complexity. For models with *r* ≳ 0.003, significant running of running is found. This means that while three free parameters (corresponding to *n*_*s*_, *α*_*s*_, *N*) were previously needed, we now need an additional free parameter. Therefore we turn to a model of a degree six polynomial potential. Obviously considering higher degree models complicates the analysis by adding other tunable parameters. The potential is given by the following polynomial:

$$V=V_{0}(1+\sum_{p=1}^{6}a_{p}\phi^{p})\;.$$ (5)

It has been shown [6] that the potential can be written as:

$$V=V_{0}(1-\sqrt{\frac{r_{0}}{8}}\phi +\frac{\eta_{0}}{2}\phi^{2}+\frac{\alpha_{0}}{3\sqrt{2r_{0}}}\phi^{3}+a_{4}\phi^{4}+a_{5}\phi^{5}+a_{6}\phi^{6}).$$ (6)

However, for simplicity, we express the potential as follows:

$$V=V_{0}(1-\sqrt{\frac{r_{0}}{8}}\phi +\sum_{p=2}^{6}a_{p}\phi^{p})\;,$$ (7)

with the subscript 0 denoting the value at the CMB point. By setting *ϕ*_0_ = 0; *ϕ*_*end*_ = 1 we limit ourselves to small field models in which Δ*ϕ* = 1 in Planck units, with little effect on CMB observables. According to the Lyth bound [19, 20], given a Tensor-to-Scalar ratio of *r* ≃ 0.01, the lower bound on the field excursion is approximately given by Δ*ϕ*_4_ ≳ 0.03 *m*_*pl*_. Here Δ*ϕ*_4_ is the field excursion while the first ∼4 efolds are generated. Our models satisfy this strict bound, as the first 4 efolds or so typically result in Δ*ϕ*_4_ ∼ 0.15 which is well above 0.03. The Lyth bound was further extrapolated [21] to cover the entire inflationary period. Applying this approach to models with *r* ∼ 0.01 yields Δ*ϕ* ≃ 2 *m*_*pl*_. However, in [7], it was shown that in models such as the ones we study, the value of Δ*ϕ* can be smaller because *ϵ*_*H*_ is non-monotonic. In this case, Δ*ϕ* = 1 *m*_*pl*_ from the CMB point to the end of inflation is consistent with the Lyth bound.

When the coefficients {*r*_0_, *a*_2_, *a*_3_, *a*_4_} are fixed, the remaining coefficients are related by:

$$a_{5}=f_{1}\left(r_{0},a_{2},a_{3},a_{4},a_{6}\right),$$ (8)

$$a_{6}=f_{2}\left(r_{0},a_{2},a_{3},a_{4},N\right).$$ (9)

The procedure of finding *f*_1_ and *f*_2_ was explained in detail for the degree 5 polynomial models in [1], and here we follow a similar procedure for the degree 6 models. So, ultimately, the model is parametrized by 5 parameters: the two physical parameters *r*_0_ and *N* and the three other parameters (*a*_2_, *a*_3_, *a*_4_) that are used to parametrize the *n*_*s*_, *α*_*s*_, *β*_*s*_ parameter space. It should be pointed out that *N* is not an observable, rather *N* ∼ 50 − 60 is a ‘soft’ constraint. Strictly speaking, *N* depends on the reheating temperature and only its maximum value can be determined. However for simplicity we treat *N* as an observable, in order to facilitate the study of a large sample of models.

## 3 Coefficient extraction methods

In this section we explain the two methods for calculating the most likely coefficients {*a*_2_, *a*_3_, *a*_4_}, given a large number of simulated models and the likelihood data for the CMB observables. This data is available through MCMC analysis of CMB data, such as the Planck data.

### 3.1 Likelihood assignment method—Gaussian extraction

To each potential, after calculating the observables *n*_*s*_, *α*_*s*_, *β*_*s*_, we assign a likelihood. For each observable we calculate the likelihood according to the CosmoMC [22] likelihood analysis of the data sets used. We then assign the product of the likelihoods $L_{\left(n_{s}\right)}\times L_{\left(\alpha_{s}\right)}\times L_{\left(\beta_{s}\right)}$ to the potential. A concrete example is the following: suppose we extract the trio (*n*_*s*_, *α*_*s*_, *β*_*s*_) = (0.96, 0.011, 0.024), we look up the likelihoods: $L_{\left(n_{s}=0.96\right)}\times L_{\left(\alpha_{s}=0.011\right)}\times L_{\left(\beta_{s}=0.024\right)}$. We now multiply them, and so the likelihood attached to that specific model which yielded these observables is given by $L_{potential}=L_{\left(n_{s}=0.96\right)}\times L_{\left(\alpha_{s}=0.011\right)}\times L_{\left(\beta_{s}=0.024\right)}$. We proceed to extract likelihoods for the different coefficients by a process of marginalization. The expectation is that this method will yield a (roughly) Gaussian distribution for each of the values of *a*_2_, *a*_3_, *a*_4_. The advantage of this method is in yielding not only the most likely value, but also the width of the Gaussian. This width can then be used as an indication for the level of tuning that is needed in these models.

#### 3.1.1 Possible pitfalls

**Fig 2:**
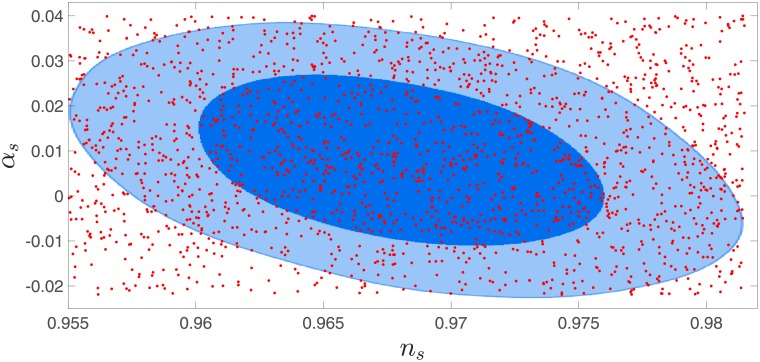
Small field inflationary potentials which yield *r* = 0.01, as well as PPS observables within 68% and 99% confidence levels. Every (*n*_*s*_, *α*_*s*_) pair is accessible using these models. The likelihood curves are results of a CosmoMC run with the latest BICEP2+Planck data.

This method of likelihood assignment is vulnerable in two ways:

a) To be valid, this method requires a uniform cover of the relevant parameter space, by the potential parameters. If the cover significantly deviates from uniform, the results might be skewed by overweighting areas of negligible weight, or underweighting areas of significant weight. Fig 2 shows a mostly uniform cover.
b) Since $L_{n_{s},\alpha_{s},\beta_{s}}≃L_{\left(n_{s}\right)}\times L_{\left(\alpha_{s}\right)}\times L_{\left(\beta_{s}\right)}$ only if the paired covariance is small, we must make sure that this is the case. In our underlying CosmoMC [22] analysis this is indeed the case. The covariance terms are, in general, one to two orders of magnitude smaller than the likelihoods at the tails of the Gaussian.
c) We also run the risk of false results if the fit we apply to the data points produced by the numerical analysis yields a large fitting error. However the fitting error of the polynomial function to the log *PPS* − log *k* data, is usually of the order of 10^−6^. This fitting is done over 30 data points generated by the MS equation numerical evaluation, for each potential. The error is calculated as $\Delta =\sqrt{\sum_{1}^{30}{\left({\left(\text{log}PPS\right)}_{i}-\text{fit}\left({\left(\text{log}k\right)}_{i}\right)\right)}^{2}}$, thus roughly speaking the error per data point is of the order of 10^−6∼7^. We conclude that the log *PPS* function is well fitted.

### 3.2 Multinomial fit

Another method for calculating the most likely coefficients is by fitting the simulated data with a multinomial function of the CMB observables. We aim to find a set of functions *F*_*i*_ such that, for example, *a*_2_ = *F*_2_(*n*_*s*_, *α*_*s*_, *β*_*s*_). We assume that this function is smooth and thus can be expanded in the vicinity of the most likely CMB observables. Hence, we can find a set of multinomials (*F*_2_, *F*_3_, *F*_4_), such that:

$$\begin{array}{c} \begin{array}{ccc} a_{2} & = & F_{2}\left(n_{s},\alpha_{s},\beta_{s}\right)\\ a_{3} & = & F_{3}\left(n_{s},\alpha_{s},\beta_{s}\right)\\ a_{4} & = & F_{4}\left(n_{s},\alpha_{s},\beta_{s}\right). \end{array} \end{array}$$ (10)

We have found that a quadratic multinomial is sufficiently accurate and that using a higher degree multinomial does not improve the accuracy significantly. Thus we may represent these by a symmetric bilinear form plus a linear term, as follows:

$$F_{i}=OB_{i}O^{†}+A_{i}O^{†}+p_{0,i}$$ (11)

where *O* = (*n*_*s*_, *α*_*s*_, *β*_*s*_), *B*_*i*_ is the bilinear matrix, and the linear coefficient vector is *A*_*i*_.

### 3.3 Pivot scale

So far, we discussed matching potentials and their resulting PPS around the CMB point. However, in order to correctly compare results of the PPS to observables, one has to take into account the pivot scale at which the CMB observables are defined. Since, in this case, the pivot scale is given by *k*_0_ = 0.05 *hMpc*^−1^, and the CMB point is at *k* ∼ 10^−4^ *hMpc*^−1^, the observables in the CMB point and *k*_0_ should be related in a simple way only if the spectrum varies slowly with *k*. This is not true for the case at hand. Two potentials can yield very different power spectra near the CMB point, and nevertheless yield the same observables at the pivot scale. These degeneracies, stem from our limited knowledge of the power spectra on small scales, and at the CMB point. For concreteness take two PPS functions, one that is well approximated by a cubic fit near the pivot scale, and the other that is well approximated only when we consider a quartic fit. Suppose, additionally, that these two PPS functions have the exact same first three coefficients, it follows that they yield the exact same observables {*n*_*s*_, *α*_*s*_, *β*_*s*_}. However if we go to sufficiently small scales, or sufficiently high *k* values, these functions will diverge. This is also true at the large scale end, where the CMB point is set. Hence the degeneracy.

A possible solution to this problem, is classifying the resulting power spectra by the level of minimal good fit. We define a good fit as one in which the cumulative relative error $\Delta =\sqrt{\sum_{k}{\left(\text{log}(PPS(\text{log}(k)))-fit(\text{log}(k))\right)}^{2}}$, is less than 10^−7^. Given a single power spectrum, we fit our result with a polynomial fit, increasing in order until the accumulated relative error is sufficiently small. The minimal degree polynomial fit that approximates the log(*PPS*) function to the aforementioned accuracy is called the minimal good fit.

We then study separately power spectra that are well fitted by cubic polynomials, quartic polynomials etc. In this way we make sure that we compare non-degenerate cases.

## 4 Monte Carlo analysis of Cosmic Microwave Background with running of running

In [11] it was shown that the inclusion of additional parameters, i.e., the running of the spectral index (*α*_*s*_), and the running of the running (*β*_*s*_) resolves much of the tension between different data sets. In this section we briefly discuss the effect of considering non-vanishing *α*_*s*_ and *β*_*s*_ on the most likely shape of the PPS. First we find *n*_*s*_ when it is the only free parameter. We then use *n*_*s*_, and *α*_*s*_ as the free parameters, and finally we conduct an analysis with *n*_*s*_, *α*_*s*_, and *β*_*s*_ as the free parameters. The shape of the power spectrum changes significantly when running of running is considered.

**Fig 3:**
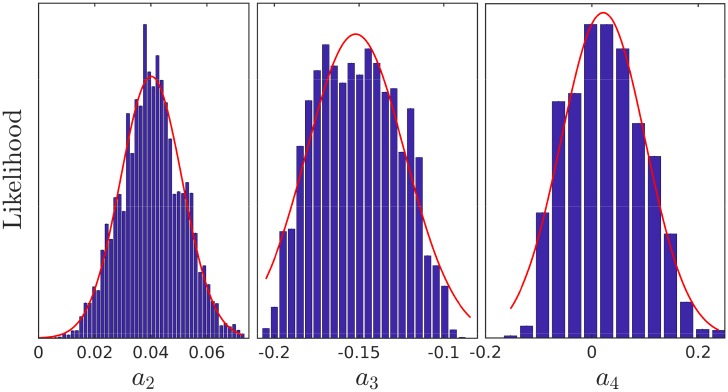
Power spectra as recoverd using CosmoMC analysis with latest BICEP2+Planck data. Allowed area (68% CL) for a fixed *n*_*s*_ analysis is shown (blue). Similarly a fixed *α*_*s*_ analysis (red), and a fixed *β*_*s*_ (green) are shown. The other colores are intersection areas. The pivot scale in this graph is at $\text{log}(\frac{k}{k_{0}})=0$, where *k*_0_ = 0.05 *hMpc*^−1^. The apparent divergence in high k’s is due to the inability of Planck to constrain these k’s. With more data, it will be possible to differentiate between the three possibilities.

**Fig 4:**
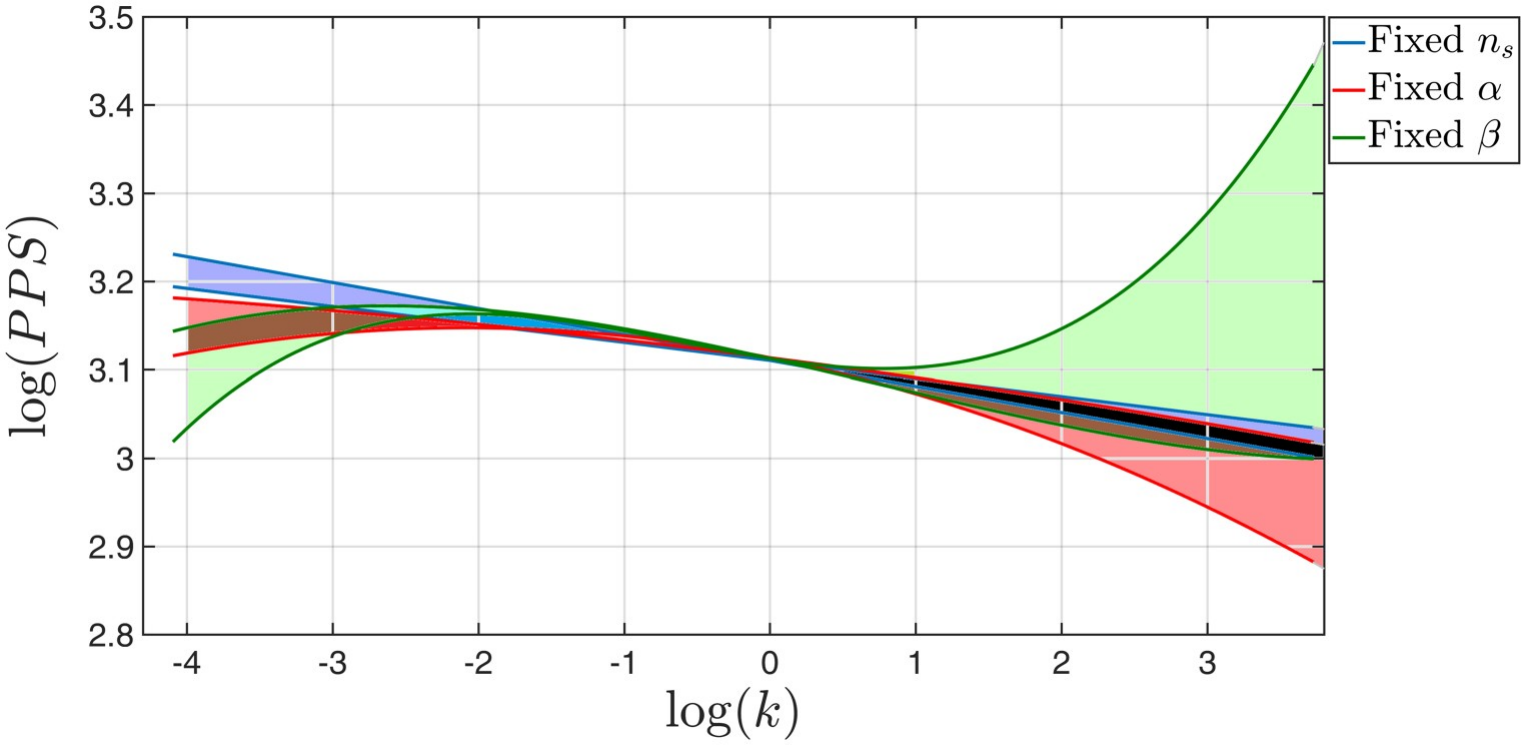
Power spectra in the *C*_*l*_’s decomposition (upper panel), with a free *n*_*s*_ (thick blue line), free *α*_*s*_ (thin cyan dots), and free *β*_*s*_ (medium red dash). The lower panel shows the realtive difference between the different cases. The relative difference (lower panel) is bound from above by ∼1%. Additionally the Planck observation error bars are shown.

The data sets that were used are the latest BICEP2+Planck baseline [15], along with the low *l*’s [23], low TEB and lensing likelihoods. The results of these analyses are given in Table 1, as well as in Fig 3. As expected the resulting power spectra converge at the pivot scale *k*_0_ = 0.05 *hMpc*^−1^. However for lower *k*’s, the resulting spectra diverge considerably, consistent with cosmic variance. Notably, the spectra also diverge at higher *k*’s. This indicates the inability of current observational data to constrain the models in this range of *k*’s. This inability is also demonstrated in Fig 4 where, for *l* > 1500, the most restrictive data cannot rule out models with significant running, or running of running. Fig 4 also shows that the three models are virtually indistinguishable in terms of the observed *C*_*l*_’s.

**Table 1:** Results from 3 analyses of the latest BICEP2+Planck dataset, each adding a free parameter in the power spectrum. The results shown are best fits, within the 68% confidence level for each analysis.

Parameter (68%)	free *n*_*s*_	free *α*_*s*_	free *β*_*s*_
log(10^10^ *A*_*s*_)	3.1047 ± 0.0057	3.1073 ± 0.006	3.1061 ± 0.0065
*n*_*s*_	0.9751 ± 0.0045	0.973 ± 0.0057	$0.9687_{-0.006}^{+0.0051}$
*α*_*s*_	N/A	−0.009 ± 0.0067	0.008±0.013
*β*_*s*_	N/A	N/A	0.020 ± 0.013

The conclusion is that we will need additional accurate data from smaller cosmic scales to be able to differentiate between the three scenarios. These extra e-folds might come from future missions such as Euclid [24], or from *μ*-type distortion data [25, 26].

## 5 Results

We apply the methods discussed in Section 3 to the degree six polynomial inflationary potentials that yield *r* = 0.01. We calculate the most likely coefficients and extract the resulting most likely polynomial inflationary potential, the results are given in Tables 2 and 3. The PPS resulting from this inflationary potential is then calculated in order to confirm that the most likely coefficients reconstruct the most likely observables.

**Table 2:** A comparison of recalculated power spectra observables from results of the two extraction methods.

Observable	Recalculated	
	Probabilistic Method	Multi-fit
*n*_*s*_	0.9687	0.9684
*α*_*s*_	0.0089	0.0076
*β*_*s*_	0.0176	0.020

**Table 3:** The most likely coefficients extracted by the process of likelihood assignment and marginalization, as well as by using the multinomial method.

	Gaussian extraction		Multinomial fit
*r* = 0.01	*μ* (average)	*σ* (standard deviation)	value
*a*_2_	0.0402	0.0156	0.01866
*a*_3_	−0.152	0.0414	−0.0235
*a*_4_	0.0215	0.1123	−0.3452

### 5.1 Results for degree six polynomials that yield *r* = 0.01

In Fig 2 we showed a cover for the joint likelihood map of *n*_*s*_ − *α*_*s*_, of about 2000 potentials with *r* = 0.01. The cover is approximately uniform, thus we were able to assign likelihoods to every potential we study, as previously discussed.

**Fig 5:**
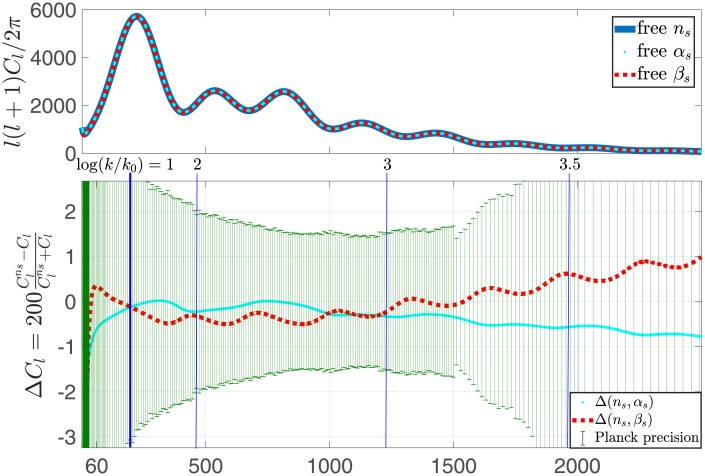
Reconstruction of the PPS from the most likely potential with *r* = 0.01, as calculated by the multinomial (reverse fitting) method (X’s and red dash), as well as the probabilistic method (circles and blue line). The CMB observables are well within the 68% confidence levels of the MCMC analysis for both. However, the probabilistic method seems to yield more precise results.

**Fig 6:**
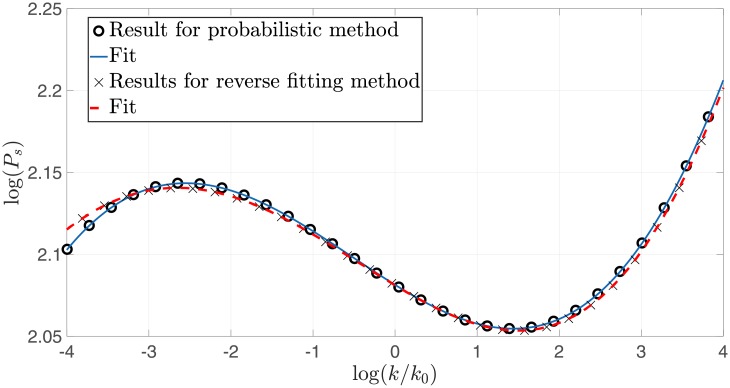
The calculated likelihoods for the coefficients {*a*_2_, *a*_3_, *a*_4_}, in models with *r* = 0.01. The most likely coefficients are given by: *a*_2_ = 0.04, *a*_3_ = −0.15, *a*_4_ = 0.02. The tuning level for each coefficient is given by Barbieri-Giudice measure and is (0.375, 0.27, 5.5).

By a process of marginalization, as discussed in Section 3, we extract the most likely coefficients, which yield the likeliest observables. This process is represented graphically in Fig 3. The results are shown in Table 3 and the PPS reconstruction is shown in Fig 5. The advantage of this method is that it also determines the deviation from the average value. This can be used as an indicator for the level of tuning that is required to construct the most likely small field model. A discussion of tuning in field theoretic models can be found in [27], as well as in [28] and [29]. In most cases the tuning level can be viewed as simply $\frac{\Delta x_{i}}{x_{i}}$, which in this case are given by (0.375, 0.27, 5.5) for *a*_2_, *a*_3_, *a*_4_ respectively. The width of the Gaussian fits for {*a*_2_, *a*_3_, *a*_4_} are {0.015, 0.041, 0.112} respectively This is shown in Fig 6. These widths represent the effective area in parameter space that yields observables within the 68% CL. Which is another measure of the tuning required in the sixth-order polynomial models.

Recalculating the CMB observables that this most likely model yields, we find *n*_*s*_ = 0.9687, *α*_*s*_ = 0.0089, *β*_*s*_ = 0.0176. These values are very close to the most likely values found from the previously discussed MCMC analysis of the BICEP2+Planck data. The resulting scalar index fits the most likely value in Table 1 exactly, while *α*_*s*_ and *β*_*s*_ deviate from these values by no more than 12.5%. We found that this is a relic of the binning method. Adding more models to the simulated data and refining the binning process results in even better proximity to the desired values.

Using the method of multinomial evaluation (3.2), we found the multinomial coefficients for each of the model degrees of freedom. For instance for *a*_2_ we have:

$$\begin{array}{c} \begin{array}{ccc} B & = & \left(\begin{array}{ccc} -20.97 & -0.936 & -37.716\\ & 19.19 & 30.402\\ & & -407.53 \end{array}\right)\\ A & = & \left(40.918,0.955,79.253\right)\\ p_{0} & = & -19.938 \end{array}. \end{array}$$ (12)

Since *n*_*s*_ ∼ 1, and *α*_*s*_ and *β*_*s*_ are of the order of 10^−2^, the above result suggests that *a*_2_ is primarily dominated by *n*_*s*_. Similarly, we have found that *a*_3_ is dominated by a linear combination of *α*_*s*_, and *β*_*s*_, and *a*_4_ is primarily dominated by *β*_*s*_. This method yields the most likely CMB observables with comparable accuracy to the previous method upon recalculation: *n*_*s*_ = 0.9684, *α*_*s*_ = 0.0077, *β*_*s*_ = 0.020. Table 2 contains a comparison between the results of the two methods.

### 5.2 Most likely potentials

Since *n*_*s*_ is better constrained, we opt for the analysis that yields a more precise value of *n*_*s*_. The leading 6th degree polynomial which yields *r* = 0.01 at the proper pivot scale, is thus given by:

$$V=V_{0}(1-0.035\phi +0.04\phi^{2}-0.15\phi^{3}+0.02\phi^{4}+0.76\phi^{5}-0.78\phi^{6}).$$ (13)

Upon initial investigation, it seems these models produce a relatively flat tensor power spectrum. This might motivate future research of the tensor power spectrum predictions and constraints.

## 6 Observable dependence

**Fig 7:**
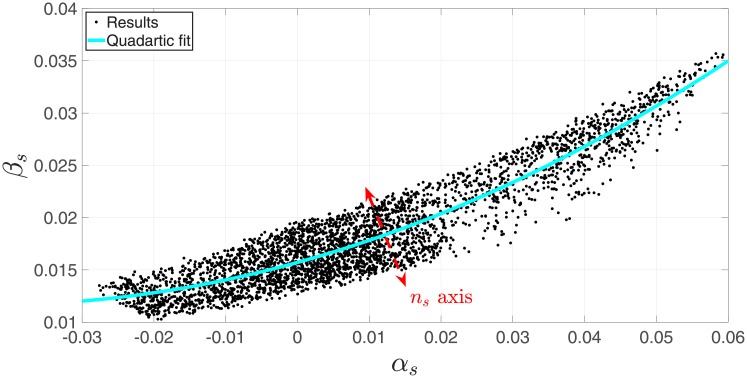
Dependence of *β*_*s*_ on the other observables, exposes an approximate quadratic relations between *α*_*s*_ and *β*_*s*_. The width of the resulting band indicates the deviation from a quadratic relation, which is correlated to *n*_*s*_.

An interesting finding is an inter-dependence of the three observables *n*_*s*_, *α*_*s*_, *β*_*s*_. For models that yield *r* = 0.01, there is a quadratic relation between the observables, such that *β*_*s*_ = *β*_*s*_(*n*_*s*_, *α*_*s*_) this is evident in Fig 7. It should be stressed that this is a phenomenon associated with the models and not with the observational data. This is supported by the small *n*_*s*_, *α*_*s*_, *β*_*s*_ paired-covariance found in [11], implying weak dependence among observables in the data itself.

One might think that the previous findings in [16, Figure 23] indicate that *n*_*s*_ and *α*_*s*_ are dependent. However, the graph shows a dependence between *α*_*s*_ and *n*_*s*,0.002_ which is the scalar index evaluated at *k* = 0.002 *hMpc*^−1^. Taking some initial *n*_*s*_ evaluated at *k*_0_, it follows that *n*_*s*_ evaluated at some other scale, depends on the index running *α*_*s*_. Indeed, when one examines the color coding in [16, Figure 23], which represents *n*_*s*_ at the pivot scale, it is clear that *n*_*s*_ and *α*_*s*_ are independent.

## 7 Summary and outlook

A large sample of potentials that yield *r* = 0.01 and conform to the allowed observable values was successfully generated. The sample provided a uniform cover of the allowed region of parameters which enabled us to assign likelihoods to each of the potentials and extract each coefficient’s likelihood (3.1). Another approach was implemented, representing each coefficient as a multinomial function of the observables (3.2), which yielded similar results. A most likely small field potential giving rise to *r* = 0.01 and (*n*_*s*_ ≃ 0.9694, *α*_*s*_ ≃ 0.009, *β*_*s*_ ≃ 0.0175), was identified, and its power spectrum simulated. An interesting inter-dependence of (*n*_*s*_, *α*_*s*_, *β*_*s*_) was found in these models, which may have some bearing on future MCMC analysis. We hope to perform such an MCMC analysis, either with priors that include this dependence or with a numerical scheme that reflects it.

The Planck collaboration may soon release additional analysis products, and BICEP3 is also expected to release results in the near future. Thus, it might be possible to check our prediction for the tensor-to-scalar ratio. However, ruling out models of the class discussed in this paper might be a more difficult task due to the lack of constraining power of current observations in the range *l* > 1500. We expect that either S4 cosmology or *μ*-distortion data will be able to resolve this in the foreseeable future by adding observational data on smaller scales.

## References

[1] Wolfson I. and Brustein R., “Small field models with gravitational wave signature supported by CMB data,” Accepted for publication in PLoS ONE, (2018) arXiv:1607.03740 [astro-ph.CO]. 10.1371/journal.pone.0197735 PMC5993128 29795608

[2] Stewart E. D. and Lyth D. H., “A More accurate analytic calculation of the spectrum of cosmological perturbations produced during inflation,” Phys. Lett. B 302, 171 (1993) [gr-qc/9302019]. 10.1016/0370-2693(93)90379-V

[3] Lyth D. H. and Riotto A., “Particle physics models of inflation and the cosmological density perturbation,” Phys. Rept. 314 (1999) 1 [hep-ph/9807278]. 10.1016/S0370-1573(98)00128-8

[4] Martin J., Ringeval C. and Vennin V., “Encyclopædia Inflationaris,” Phys. Dark Univ. 5-6 (2014) 75 [arXiv:1303.3787 [astro-ph.CO]]. 10.1016/j.dark.2014.01.003

[5] Dodelson S. and Stewart E., “Scale dependent spectral index in slow roll inflation,” Phys. Rev. D 65 (2002) 101301 [astro-ph/0109354]. 10.1103/PhysRevD.65.101301

[6] Ben-Dayan I. and Brustein R., “Cosmic Microwave Background Observables of Small Field Models of Inflation,” JCAP 1009, 007 (2010) [arXiv:0907.2384 [astro-ph.CO]]. 10.1088/1475-7516/2010/09/007

[7] Hotchkiss S., Mazumdar A. and Nadathur S., “Observable gravitational waves from inflation with small field excursions,” JCAP 1202, 008 (2012) [arXiv:1110.5389 [astro-ph.CO]]. 10.1088/1475-7516/2012/02/008

[8] Choudhury S. and Mazumdar A., “Reconstructing inflationary potential from BICEP2 and running of tensor modes,” arXiv:1403.5549 [hep-th].

[9] Chatterjee A. and Mazumdar A., “Bound on largest *r* ≲ 0.1 from sub-Planckian excursions of inflaton,” JCAP 1501 (2015) no.01, 031 [arXiv:1409.4442 [astro-ph.CO]]. 10.1088/1475-7516/2015/01/031

[10] Choudhury S., “Reconstructing inflationary paradigm within Effective Field Theory framework,” Phys. Dark Univ. 11 (2016) 16 [arXiv:1508.00269 [astro-ph.CO]]. 10.1016/j.dark.2015.11.003

[11] Cabass G., Di Valentino E., Melchiorri A., Pajer E. and Silk J., “Constraints on the running of the running of the scalar tilt from CMB anisotropies and spectral distortions,” Phys. Rev. D 94, no. 2, 023523 (2016) [arXiv:1605.00209 [astro-ph.CO]]. 10.1103/PhysRevD.94.023523

[12] Cortes M., Liddle A. R. and Mukherjee P., “On what scale should inflationary observables be constrained?,” Phys. Rev. D 75, 083520 (2007) [astro-ph/0702170].

[13] Liddle A. R., Parkinson D., Leach S. M. and Mukherjee P., “The WMAP normalization of inflationary cosmologies,” Phys. Rev. D 74 (2006) 083512 [astro-ph/0607275].

[14] Peiris H. and Easther R., “Slow Roll Reconstruction: Constraints on Inflation from the 3 Year WMAP Dataset,” JCAP 0610 (2006) 017 [astro-ph/0609003]. 10.1088/1475-7516/2006/10/017

[15] Ade P. A. R., Aghanim N., Ahmed Z., Aikin R. W., Alexander K. D., Arnaud M. et al [BICEP2 and Planck Collaborations], “Joint Analysis of BICEP2/*Keck Array* and *Planck Data*,” Phys. Rev. Lett. 114, 101301 (2015) [arXiv:1502.00612 [astro-ph.CO]]. 10.1103/PhysRevLett.114.101301 25815919

[16] Ade P. A. R., Aghanim N., Arnaud M., Ashdown M., Aumont J., Baccigalupi C. et al [Planck Collaboration], “Planck 2015 results. XIII. Cosmological parameters,” Astron. Astrophys. 594 (2016) A13 [arXiv:1502.01589 [astro-ph.CO]]. 10.1051/0004-6361/201525830

[17] Yamaguchi M., “Supergravity based inflation models: a review,” Class. Quant. Grav. 28, 103001 (2011) [arXiv:1101.2488 [astro-ph.CO]]. 10.1088/0264-9381/28/10/103001

[18] Baumann D. and McAllister L., “Inflation and String Theory,” arXiv:1404.2601 [hep-th].

[19] Lyth D. H., “What would we learn by detecting a gravitational wave signal in the cosmic microwave background anisotropy?,” Phys. Rev. Lett. 78 (1997) 1861 [hep-ph/9606387]. 10.1103/PhysRevLett.78.1861

[20] Easther R., Kinney W. H. and Powell B. A., JCAP 0608 (2006) 004 [astro-ph/0601276].

[21] Efstathiou G. and Mack K. J., “The Lyth bound revisited,” JCAP 0505 (2005) 008 [astro-ph/0503360]. 10.1088/1475-7516/2005/05/008

[22] Lewis A. and Bridle S., “Cosmological parameters from CMB and other data: A Monte Carlo approach,” Phys. Rev. D 66, 103511 (2002) [astro-ph/0205436]. 10.1103/PhysRevD.66.103511

[23] Bennett C. L., Larson D., Weiland J. L., Jarosik N., Hinshaw G., Odegard N. et al [WMAP Collaboration], “Nine-Year Wilkinson Microwave Anisotropy Probe (WMAP) Observations: Final Maps and Results,” Astrophys. J. Suppl. 208,20 (2013) [arXiv:1212.5225 [astro-ph.CO]]. 10.1088/0067-0049/208/2/20

[24] Amendola L., Appleby S., Avgoustidi A., Bacon D., Baker T., Baldi M. et al, “Cosmology and Fundamental Physics with the Euclid Satellite,” arXiv:1606.00180 [astro-ph.CO]. 10.1007/s41114-017-0010-3 PMC5897888 29674941

[25] Diacoumis J. A. D. and Wong Y. Y. Y., “CMB spectral distortions as a novel way to probe the small-scale structure problems,” arXiv:1710.03121 [astro-ph.CO].

[26] Abitbol M. H., Chluba J., Hill J. C. and Johnson B. R., “Prospects for Measuring Cosmic Microwave Background Spectral Distortions in the Presence of Foregrounds,” Mon. Not. Roy. Astron. Soc. 471 (2017) 1126 [arXiv:1705.01534 [astro-ph.CO]]. 10.1093/mnras/stx1653

[27] Barbieri R. and Giudice G. F., “Upper Bounds on Supersymmetric Particle Masses,” Nucl. Phys. B 306 (1988) 63 10.1016/0550-3213(88)90171-X

[28] Ellis J. R., Enqvist K., Nanopoulos D. V. and Zwirner F., “Observables in Low-Energy Superstring Models,” Mod. Phys. Lett. A 1 (1986) 57.

[29] Fowlie A., “CMSSM, naturalness and the “fine-tuning price” of the Very Large Hadron Collider,” Phys. Rev. D 90 (2014) 015010 [arXiv:1403.3407 [hep-ph]]. 10.1103/PhysRevD.90.015010

